# Characteristics of the Cross-Sectional Vorticity of the Natural Spawning Grounds of *Schizothorax prenanti* and a Vague-Set Similarity Model for Ecological Restoration

**DOI:** 10.1371/journal.pone.0136724

**Published:** 2015-08-28

**Authors:** Ming-Yang Liu, Ling-Lei Zhang, Jia Li, Yong Li, Nan Li, Ming-Qian Chen

**Affiliations:** 1 Institute of Ecology and Environment, State Key Laboratory of Hydraulics and Mountain River Engineering, College of Water Resource & Hydropower, Sichuan University, Chengdu, 610065, Sichuan, China; 2 School of Economics, Sichuan University, Chengdu, 610065, Sichuan, China; 3 Water Bureau of Dazhou city, No. 616 West Ring Road, Dazhou 635000, Sichuan, China; 4 Sichuan Environment & Engineering Appraisal Center, Chengdu, Sichuan, China; University of Aveiro, PORTUGAL

## Abstract

*Schizothorax prenanti* is an endemic fish in the mountain rivers of southwestern China with unique protection value. To further explore the vortex motion of hydraulic habitats, which is closely related to the fish breeding process, the cross-sectional vorticity was used to evaluate the hydraulic conditions of the natural spawning habitat of *S*. *prenanti*. A coupled level-set and volume-of-fluid (CLSVOF) three-dimensional (3D) model was applied to simulate the hydraulic habitat of the Weimen reach, a typical natural spawning ground for *S*. *prenanti* in the upper Yangtze River. The model was used in conjunction with the Wilcoxon rank sum test to distinguish the distributions of vertical vorticity in spawning and non-spawning reaches. Statistical analysis revealed that the cross-sectional vorticity in spawning reaches was significantly greater than in non-spawning reaches, with likely biological significance in the spawning process. The range of cross-sectional mean values of vorticity was 0.17 s^-1^–0.35 s^-1^ in areas with concentrated fish sperm and eggs; the minimum value was 0.17 s^-1^, and the majority of values were greater than 0.26 s^-1^. Based on this study, a vague-set similarity model was used to assess the effectiveness of ecological restoration by evaluating the similarity of the cross-sectional vorticity of the natural spawning reach and rehabilitated spawning reach after implementing ecological restoration measures. The outcome might provide a theoretical basis for the recovery of damaged *S*. *prenanti* spawning grounds and act as an important complement for the assessment of recovery effectiveness and as a useful reference for the coordination of ecological water use with the demands of hydraulic and hydropower engineering.

## Introduction

*Schizothorax prenanti*, a semi-migratory fish endemic to China that matures in inland waters, is a Class-II provincial protected animal and an important commercial fish that inhabits the upper reaches of the Yangtze River and tributaries such as the Jinsha, Dadu, Minjiang, and Qingyi [1]. The rapid growth of the social economy has led to the construction of many water conservation and hydropower works that have caused a series of ecological problems. The construction of multistage dams, including the Jiyu, Tongzhong, Jiangshe, Zhongba, Futang, Taipingyi, Yingxiuwan and Zipingpu dams, has blocked the migration route of fish to the upper reaches of the Minjiang River. Furthermore, the dams have likely destroyed the hydraulic environment of the spawning grounds required by *S*. *prenanti*, directly decreasing spawning rates [2, 3].

Ideally, a river should have a variety of native aquatic organisms and an abundance of specific habitats to sustain the different life stages of fish. Unfortunately, dams, diversion projects and many other human activities in and around rivers drastically alter and damage habitat conditions within a river, causing a significant decline in the biological diversity of aquatic ecosystems [4–6]. Spawning grounds, which support breeding, are believed to be the most essential and sensitive habitats for fish. Unique hydraulic characteristics are exhibited in fish spawning grounds and may have important ecological significance for the reproduction process of fish. A series of traditional hydraulic variables have been used to describe the spawning sites of fish. Jowett [7] indicated that the velocity/depth ratio and Froude number may be the best determinants of habitat type and show the most significant differences between habitat types. Sempeski and Gaudin [8], Armstrong et al. [9], Louhi et al. [10] and Rosenfeld et al. [11] confirmed that spawning habitats are characterized by a particular water depth and current velocity. Lamourou et al. [12] and Moir et al. [13, 14] suggested that local hydraulic metrics influence spawning habitats with varying importance and indicated that the Reynolds number or Froude number may be useful single descriptors of hydraulic habitat because they are dimensionless and thus comparable between different river sizes and fish species. Nevertheless, these measurements fall short of a full description of the hydraulic habitats because the spatial variation of the complex flow fields has not been described for spawning grounds.

Because of the dual complexity of fish behavior and river morphology, intensive efforts are underway to study the spawning behavior and hydraulic habitats of fish. Most fish species are known to copulate and spawn in areas of rolling and upwelling water because such water currents provide both eggs and offspring with sufficient oxygen and steady water temperatures and produce a favorable flow pattern for the spread and adhesion of fertilized eggs in gravel and rocks [15–18]. The so-called ‘rolling and upwelling water’ phenomenon is interesting and has positive biological and profound hydraulic significance. To more accurately quantify the optimal spatial flow features for fish, researchers have analyzed the advantages of using more intricately detailed physical variables to evaluate the complex flow patterns in spawning habitats. Vehanen et al. [19] demonstrated that environmental gradients (canonical correspondence analysis, with water velocity and depth) play a key role in flow regulation of fish communities. Within the context of river disturbance, the role of gradients provides an example of the longitudinal effects of flow regulation on the composition of fish assemblages and temporal changes in fish abundance and habitat use. Crowder and Diplas [20–22] proposed and tested the use of certain spatial hydraulic metrics, including local velocity gradients, kinetic energy gradients, vorticity and circulation, to quantify flow complexity occurring within micro-, meso-, and macro-habitat features and relate the metrics to the habitats that fish utilize. All of these metrics might be necessary to describe the important physical attributes of a particular location within a river, and they provide a useful method of differentiating between locations with similar depth and velocity that are surrounded by different flow patterns; however, the vorticity metric exhibited increased sensitivity in the detection of spatially varying flow patterns that produce large absolute values in complex flows and small absolute values in uniform flows [21–22]. Furthermore, Bandyopadhyay et al. [23] observed that the vortices influenced by the movement of the fins are crucial for the maneuverability of fish. Hanke et al. [24] reported that piscivorous predators and copepods detect the presence of prey through vortex structures left by swimming fish and other organisms. In an investigation of the preferred hydraulic and morphologic conditions for the spawning of Chinese sturgeon, Fu et al. [25, 26] reported that the flow fields of vortices at the bottom of holes are markedly beneficial for fish egg sedimentation. Yang et al. [27–29] and Wang et al. [30–32] demonstrated that the vorticity metric is a sensitive measurement that can describe the hydraulic characteristics of Chinese sturgeon spawning grounds, and the vorticity strength selection of Chinese sturgeon spawning indicates that larger eddies strengthen sperm-egg fusion and enhance fertilization rates. Similarly, Zhu et al. [33] confirmed that the spatial variability characteristics of the vorticity values could provide hydrodynamic indicators for spawning grounds of the four major Chinese carps in the Yangtze River middle reach. Thus, fish utilize vortices for diverse purposes in a variety of manners at different scales, and vortex motion plays a crucial role in the life processes of fish, especially fish breeding.

Limited efforts have been undertaken to protect and restore river habitats and characterize habitat requirements [9, 10, 14, 21, 22, 30, 34–46]. Whether these protection and restoration efforts are effective is dependent on establishing appropriate habitat suitability criteria for particular organisms, and the appropriateness of the criteria is dependent on ascertaining meaningful metrics. Previous work has validated metrics and evaluated habitat suitability for the spawning habitats of certain fish species around the world, particularly Atlantic salmon (*Salmo salar*) [9, 10, 13, 17], brown trout (*Salmo trutta*) [9, 10, 16], Chinook salmon (*Salvelinus fontinalis*) [14, 16], and Chinese sturgeon (*Acipenser sinensis*) [25–32, 39]. Based on quantitative analyses of endangerment level, degree of genetic loss and species value, *S*. *prenanti* has been identified as a second-level threatened and priority-protected species among the dozens of endemic fishes in the upper reaches of the Yangtze River [47]. However, a paucity of research has been conducted to quantify flow complexity and relate such complexity to the suitable habitats of *S*. *prenanti*. In addition, previous studies have indicated that vorticity analyses are a relatively new and valuable tool for describing spawning habitats. Indeed, studies on the vorticity characteristics and vorticity suitability of spawning habitats are still rare and in exploratory stages, even for fish species that have received intensive attention.

Vortex motion is one of the most common forms of fluid movement, and it is usually described by vorticity [21, 48]. Vorticity is a measure of the local spin of a fluid element or the microscopic measure of rotation at a point in a fluid. In a study investigating vorticity characteristics and their vital linkage with spawning grounds, Crowder and Diplas [21] used a two-dimensional (2D) hydraulic model to reproduce the localized complex flow features and suggested that horizontal vorticity could provide a method of quantifying spatial flows; however, because of model limitations, they did not consider vertical vorticity, i.e., vorticity around the stream-wise axis (its mean value in cross-section is frequently expressed as the cross-sectional mean vorticity or the cross-sectional vorticity in the present article). Wang et al. [30–32] applied the Delft3D-Flow model to simulate the hydraulic suitability of spawning grounds downstream of the Gezhouba Dam for Chinese sturgeon and calculated the horizontal mean vorticity values, which was significantly correlated with unit-area egg density. Yang et al. [27–29] and Zhu et al. [33] both utilized flow field data, determined using the Acoustic Doppler Current Profiler (ADCP), to calculate the cross-sectional mean vorticity; the former found that the spawn density per unit area is positively related to the cross-sectional vorticity of Chinese sturgeon spawning sites, and the latter indicated that the spatial variability characteristics of the cross-sectional vorticity can be quantified as a visual description of flow conditions on the major spawning grounds of carp. Both horizontal and vertical vorticity are important variables [29], and vorticity and fish-egg density are strongly coupled, regardless of the orientation.

In recent years, our research team has been devoted to studying the hydraulic characteristics of the spawning grounds of *S*. *prenanti*. Based on the physical structure attributes and ecological significance of hydraulic habitats, Chen et al. [43, 44] proposed a hydraulic index system composed of a geometric characteristic variable (depth), flow kinematic variables (velocity, velocity gradient, and vorticity) and dynamic variables (Froude number and kinetic energy gradient) to produce a relatively comprehensive description of the hydraulic characteristics of the spawning grounds of *S*. *prenanti*. In addition to the preliminary establishment of an index database, the advisable interval and suitability curves of each index were also detailed in this hydraulic index system. By utilizing the index system, Chen et al. [43] and Liu et al. [45, 46] have evaluated the effectiveness of riverbed modifications (gradient adjustments and section morphology changes) and hydraulic structures (ecological spur dikes) in restoring the spawning grounds of *S*. *prenanti*. However, the vorticity in this index system only referred to the horizontal vorticity and did not include the vertical vorticity because of limitations in the applied 2D hydraulic model.

To further examine vorticity and improve our current index system, the present study examines the cross-sectional vorticity of the spawning grounds of *S*. *prenanti* through field investigations, numerical simulations, rank sum tests and vague set analyses. To our knowledge, information on the cross-sectional vorticity characteristics and distribution of the spawning grounds of *S*. *prenanti* has not been previously reported. This article expands on the study objectives and computational methods of previous similar research. The relationship between the spawning grounds of *S*. *prenanti* and cross-sectional mean vorticity is analyzed, and the vorticity similarity vague-set model is proposed as a new model. The results of this study will provide a new index for our existing hydraulic database and a theoretical foundation for the restoration of *S*. *prenanti* spawning grounds, the protection of biodiversity in the region, and an improved understanding of the hydraulic characteristics and rehabilitation of other fish habitats. Moreover, because the ecological environments of rivers continue to degrade with the development of cascade hydropower, this study may help coordinate between ecological water requirements and hydropower station demands.

## Materials and Methods

### Study Area

Based on perennial observations and analyses by aquatic scientists, a few natural spawning grounds for *S*. *prenanti* have been located in the upper reaches of the Minjiang River (from the Heishui River intersection to Mao County); these areas have retained natural channel morphology, with a high heterogeneity of flow patterns. The Weimen spawning ground was affirmed as one of these scarce habitats [43] and was thus selected as a typical area for the study. The stretch of river consists of spawning, non-spawning and transition areas and has a length of 1,156 m, a width of 45.3–81.6 m, a water depth of 0–5.96 m, and a reach longitudinal gradient of approximately 1%. Related data on hydrology and terrain were obtained from detailed historical surveys and field measurements. Due to limited conditions, the vorticity values quantified in this work are representative of the flow conditions modeled within the Weimen reach and the average discharge during the spawning season of *S*. *prenanti*.

### Numerical Simulations

#### CLSVOF model

The coupled level-set and volume-of-fluid (CLSVOF) three-dimensional (3D) model available in Fluent 14.5 (ANSYS Inc., USA) was applied to simulate the flow field within the Weimen section. The velocity flow data for the horizontal mean vorticity computations were obtained by CLSVOF-Flow simulation using the model. The level-set (LS) function *φ* is defined as a signed distance to the interface. Accordingly, the interface is the zero LS, and *φ*(*x*, *t*) is expressed as Γ = {x | *φ*(*x*, *t*) = 0} in a two-phase flow system as follows:

$$\varphi (\vec{x},t)=\left\{ \begin{array}{l} +\left|d\right|\text{ if }\vec{x}\in \text{the primary interface}\\ 0\text{ if }\vec{x}\in \Gamma \\ -\left|d\right|\text{ if }\vec{x}\in \text{ the secondary interface} \end{array}\right.$$ (1)

where $\vec{x}$ is the position of the interface and *d* is the distance of the interface.

The normal ($\vec{n}$) and the curvature (*κ*) of the interface, which are required in the computation of the surface tension force, are determined as follows:

$$\vec{n}=\frac{\nabla \varphi }{\left|\nabla \varphi \right|}\;and\;\kappa =\nabla \cdot \frac{\nabla \varphi }{\left|\nabla \varphi \right|}$$ (2)

where ᐁ is the gradient operator.

The zero LS contours represent the interface, and all LS values are located at the center of the cell and are assigned as the shortest distance to the interface. The LS function is initialized as a distance function because of its important property, namely,|ᐁ*φ*| = 1, which is used to make a number of simplifications.

After initialization, the LS function is moved with the flow field, according to the following advection equation:

$$\frac{\partial \varphi }{\partial t}+\left(\vec{u}\cdot \nabla \right)\varphi =0$$ (3)

where $\vec{u}$ is the underlying velocity in the 3D model.

In the volume-of-fluid (VOF) method, the interface is tracked using the VOF function *F*, which is defined as the liquid volume fraction in a cell with values between zero and one in a surface cell and values of zero and one in air and liquid, respectively. The VOF functions are advanced by the following propagating equation:

$$\frac{\partial F}{\partial t}+\left(\vec{u}\cdot \nabla \right)F=0$$ (4)

The momentum equation can be written as follows:

$$\frac{\partial \left(\rho \vec{u}\right)}{\partial t}+\nabla \cdot \left(\rho \vec{u}\vec{u}\right)=-\nabla p+\nabla \cdot \mu \left[\nabla \vec{u}+{\left(\nabla \vec{u}\right)}^{T}\right]-\sigma \kappa \delta \left(\varphi \right)\nabla \varphi +\rho \vec{g}$$ (5)

From this equation, *δ*(*φ*) is the distance function and is defined as follows:

$$\delta \left(\varphi \right)=\{\begin{array}{ll} \frac{1+\text{cos}\left(\pi \varphi /\alpha \right)}{2\alpha } & \left|\varphi \right|<\alpha \;and\;\alpha =1.5h\\ 0 & \left|\varphi \right|\geq \alpha \end{array}$$ (6)

where *p* is the pressure, *h* is the minimum size of the cell, and *σ* is the surface tension coefficient.

After reconstruction of the interface, the LS functions must be re-distanced to achieve mass conservation. The re-distancing of the LS functions includes the initial determination of the sign of the LS function and the subsequent calculation of the shortest distance from the cell centers to the reconstructed interface via a geometric process.

#### The cross-sectional mean vorticity model

**Fig 1:**
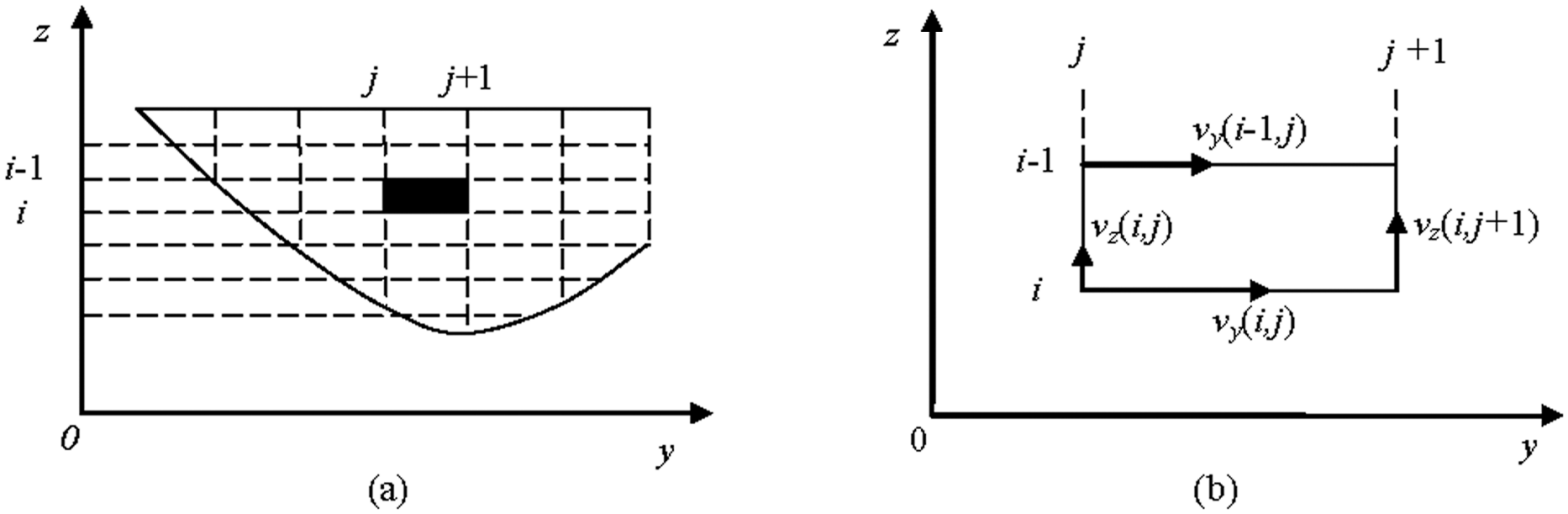
Schematic illustration of the computational process for cross-sectional mean vorticity strength. (a) Cross-sectional velocity distribution in the flow model; (b) Computational process for the velocity vector of the cell highlighted in black in (a).

The cross-sectional mean vorticity is defined as follows:

$$\bar{\Omega }=\frac{\Gamma }{A_{TOT}}=\frac{\underset{S}{\iint }\Omega \Delta A}{A_{TOT}}=\frac{\Sigma \Omega \Delta A}{A_{TOT}}=\frac{\Sigma \left|\left[\frac{\Delta v_{z}}{\Delta y}-\frac{\Delta v_{y}}{\Delta z}\right]\right|\Delta y\Delta z}{\Sigma \Delta y\Delta z}$$ (7)

where $\bar{\Omega }$ is the cross-sectional mean vorticity, *A*_*TOT*_ is the wetted cross-sectional area along a transect, Γ is the circulation parameter, Ω is the vorticity around the x-axis as computed from a cell, Δ*ν*_*z*_ is the change in the z-velocity component along the y-coordinate, Δ*ν*_*y*_ is the change in the y-velocity component along the z-coordinate, and Δ*y* and Δ*z* are the changes in distance corresponding to the above changes. Fig 1 illustrates these variables (in the coordinate system, *x* represents the stream-wise coordinate, *y* represents the transverse coordinate, *z* represents upward and normal to the *x*-*y* plane, *ν*_*y*_ is the component of the flow velocity $\vec{v}$ perpendicular to the gravitational vector, and *ν*_*z*_ is the component of the flow velocity $\vec{v}$ parallel to the gravitational vector).

According to the figure, the vorticity strength on the *y*- and *z*-coordinates is obtained as follows:

$$\frac{\Delta v_{z}}{\Delta y}=\frac{v_{z}{}_{{}_{i,j+1}}-v_{z}{}_{{}_{i,j}}}{y_{i,j+1}-y_{i,j}},\frac{\Delta v_{y}}{\Delta z}=\frac{v_{y}{}_{{}_{i-1,j}}-v_{y}{}_{{}_{i,j}}}{z_{i-1,j}-z_{i,j}}$$ (8)

#### Boundary Conditions

The spawning period of *S*. *prenanti* primarily occurs in April in the study area [1, 43]; thus, the boundary conditions in the numerical simulation were set according to measured data from April. The discharge at which the hydraulic model was run was consistent with an average flow for the typical spawning season and representative of a natural base flow in the study reach. The average velocity ($\bar{u}$) of the entrance boundary conditions was 1.57 ms^-1^, which was obtained from the inlet discharge and the cross-sectional area at the inlet. The free surface was set to the pressure inlet. The water surface elevations of the inlet and outlet were 1,587.76 m and 1,583.63 m, respectively. The outlet was set to the pressure outlet. A no-slip and stationary wall was adopted as the channel bed and banks using a wall function method following Versteeg and Malalasekera [49]. The initial turbulent kinetic energy and turbulent dissipation rate were calculated as $k=0.00375\;{\bar{u}}^{2}$ and *ε* = *k* ^3/2^ / (0.4*h*), respectively, where *h* is the depth of the inlet cross-section (*h* = 1.15 m).

#### Mesh Construction

**Fig 2:**
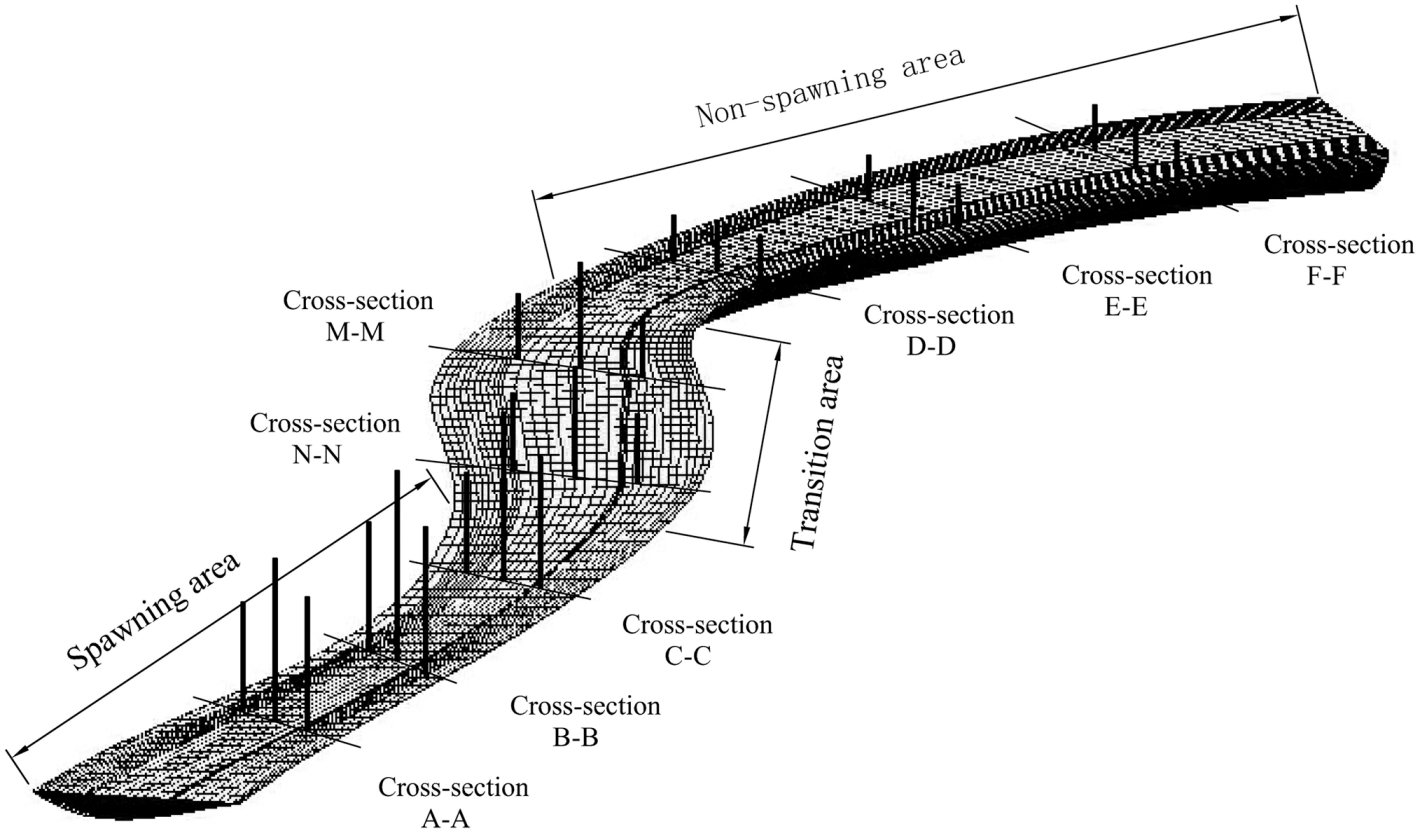
Computational mesh and typical cross-section positions in the Weimen reach.

The computational domain was discretized to obtain a total grid mesh of 12,722,562 unstructured 3D cells. Computational mesh and typical cross-sectional positions in the Weimen reach of the upper Minjiang River are shown in Fig 2, with A-A, B-B and C-C cross-sections in the spawning area, N-N and M-M cross-sections in the transition area, and D-D, E-E and F-F cross-sections in the non-spawning area.

#### Model Verification

Our previous research [50, 51] demonstrated that the 3D *k-ε* turbulence model coupled with the VOF model of the free surface problem could simulate the flow complexity preferred by *S*. *prenanti* because the computational results are consistent with experimental data. Mark and Elbridge [52] presented the CLSVOF method for computing 3D and axisymmetric incompressible two-phase flows and verified that the CLSVOF method is superior to both standalone VOF and LS. A number of studies [53–55] later applied the CLSVOF method to calculate various complicated flow fields and obtained satisfactory results that were consistent with field-measured and model-simulated data. Based upon prior testing of the CLSVOF 3D model, we assume that the model will work well in this particular scenario. The CLSVOF model simulation was consequently verified as suitable for this study, and the outputs were acceptable.

### Data Analyses

To further identify the distribution of vorticity values computed from all the individual cells in the study reaches, the Wilcoxon rank sum test was used to compare spawning reaches with non-spawning reaches by testing the differences between the overall distribution functions *F*(*x*) and *F*(*y*). We tested the null hypothesis that *F*(*x*) and *F*(*y*) are equal in distribution against the alternative that *F*(*y*) is stochastically strictly not equal to *F*(*x*):

$$H_{0}:F(x)=F(y);H_{1}:F(x)\neq F(y)$$ (9)

The original hypothesis, *H*_0_, was that the vorticity distributions of the spawning sections and non-spawning sections are the same. The alternative hypothesis, *H*_1_, was that the distributions differ. The data for three vertical cross-sections (*V*−*Vor*_*A*_, *V*−*Vor*_*B*_, *V*−*Vor*_*C*_) constituted the sampling group for the spawning area, and the data for three vertical cross-sections (*V*−*Vor*_*D*_, *V*−*Vor*_*E*_, *V*−*Vor*_*F*_) constituted the sampling group for the non-spawning area. The Wilcoxon test was used to evaluate the two groups of values at a significance level of 5%. The assumption in the control process was as follows:

$$H_{0}:F_{x}(V-Vor_{A},V-Vor_{B},V-Vor_{C})=F_{y}(V-Vor_{D},V-Vor_{E},V-Vor_{F})$$ (10)

$$H_{1}:F_{x}(V-Vor_{A},V-Vor_{B},V-Vor_{C})\neq F_{y}(V-Vor_{D},V-Vor_{E},V-Vor_{F})$$ (11)

Detailed testing and calculation methods were derived from Capitani and Martini [56], Taheri and Hesamian [57], and Moore and Chaplin [58].

### Vague Sets

The vague sets were proposed to establish the similarity model to guide ecological restoration and conducted following Li et al. [59].

## Results

### Vorticity Distribution

**Fig 3:**
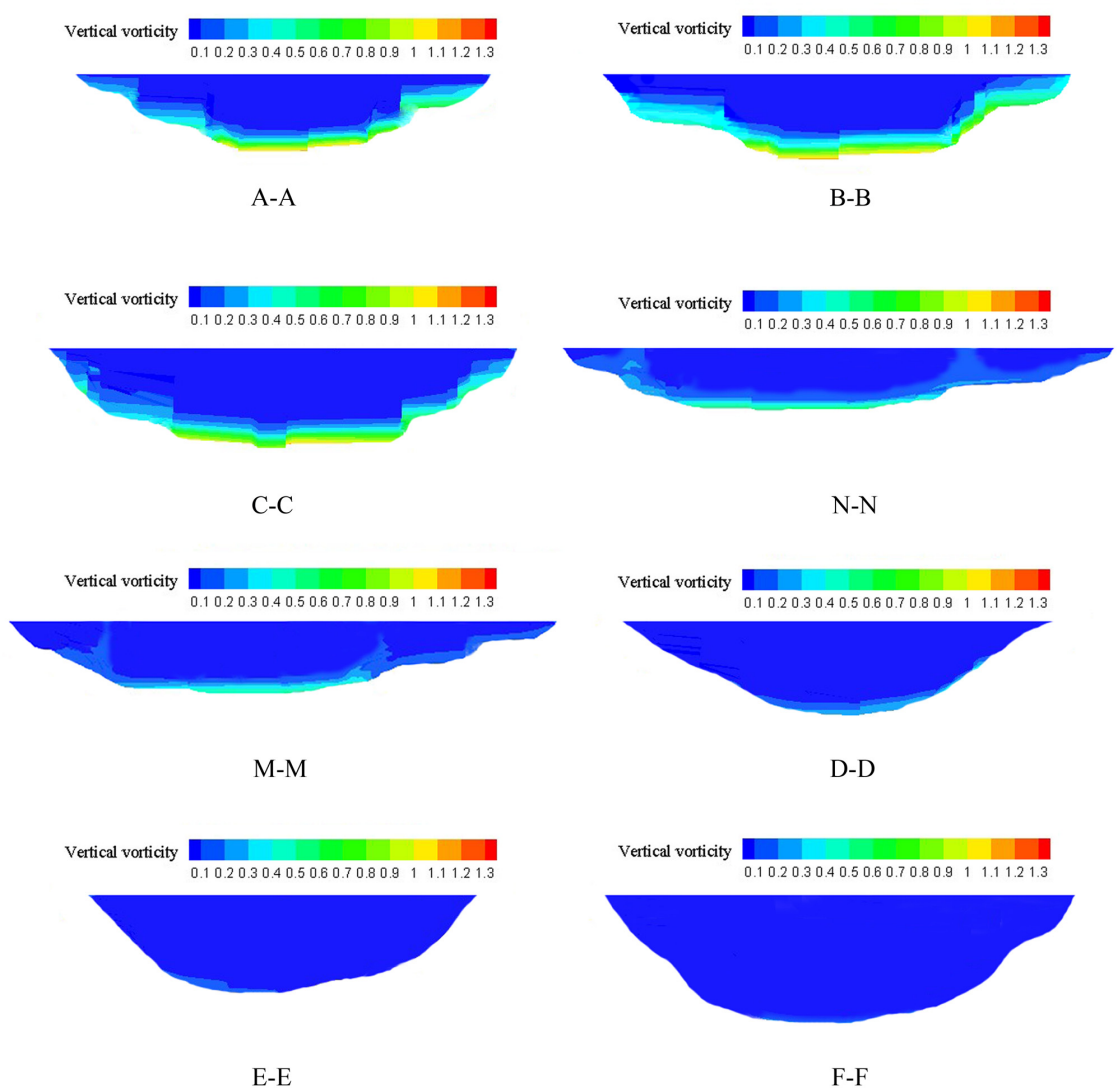
Spatial distribution of the vertical vorticity strength in various sections (s^-1^).

We can obtain the distribution of vorticity by selecting three typical cross-sections in spawning and non-spawning river areas and comparing their hydraulic habitat characteristics. The spatial distributions of the vorticity strength in the various sections (shown in Fig 3) varied between areas and were more dispersed in spawning areas. The strength of the vertical vorticities in the spawning area were greater than that in the non-spawning and transition areas. High-intensity vertical vorticity generally occurred near the riverbed bottom, and the vorticities in the spawning areas with values greater than 0.1 s^-1^ were distributed from the river bottom to 1.3 m above the riverbed. In addition, low-intensity vertical vorticity appeared at the river surface.

The statistical data in Tables 1 and 2 indicate that the influence of topography on the cross-sectional vorticity distribution differs considerably among different areas, as illustrated in Fig 3. The mean vorticity of typical cross-sections in spawning areas ranged primarily from 0.227 to 0.305 s^-1^, and in non-spawning areas, it was approximately 0.100 s^-1^. The mean vorticity strength was maximal in cross-section B-B, with values of 0.339 s^-1^ from the bottom of the river to 1.3 m above the riverbed and 0.305 s^-1^ throughout the entire cross-section, and minimal in cross-section F-F, with a value of 0.10001 s^-1^ both at the bottom of the river and throughout the cross-section.

**Table 1:** Cross-sectional mean vorticity from the river bottom to 1.3 meters above the riverbed.

Regional division	Spatial distribution of vorticity strength in the corresponding spawning area (s^-1^)				
	Section No.	Left	Middle	Right	Mean
Spawning area	A-A	0.23973	0.35652	0.29233	0.29619
	B-B	0.28246	0.41803	0.31754	0.33934
	C-C	0.22054	0.37016	0.28911	0.29327
Transition area	N-N	0.17135	0.24331	0.15274	0.18913
	M-M	0.14402	0.23257	0.13245	0.16968
Non-spawning area	D-D	0.10002	0.11131	0.10034	0.10389
	E-E	0.10001	0.10101	0.10001	0.10034
	F-F	0.10001	0.10001	0.10001	0.10001

**Table 2:** Cross-sectional mean vorticity of the entire sections.

Regional division	Spatial distribution of vorticity strength in the corresponding spawning area (s^-1^)				
	Section No.	Left	Middle	Right	Mean
Spawning area	A-A	0.21135	0.27012	0.30157	0.26101
	B-B	0.27951	0.33086	0.30378	0.30471
	C-C	0.18927	0.26458	0.22615	0.22667
Transition area	N-N	0.16366	0.21985	0.14781	0.17711
	M-M	0.14006	0.20174	0.12988	0.15723
Non-spawning area	D-D	0.10001	0.10003	0.10001	0.10002
	E-E	0.10001	0.10001	0.10001	0.10001
	F-F	0.10001	0.10001	0.10001	0.10001

**Fig 4:**
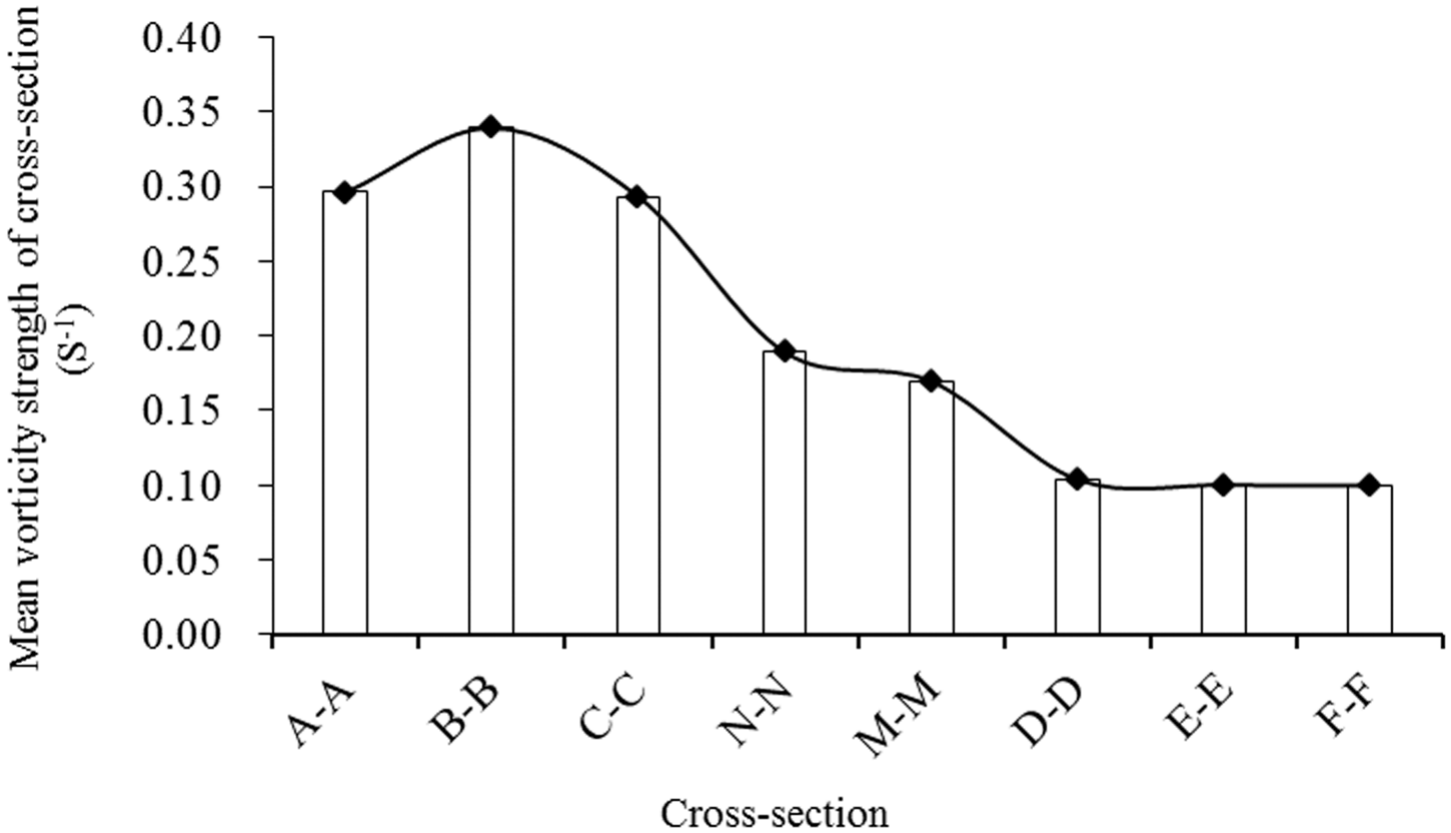
Changes of cross-sectional mean vorticity from the bottom of the river to 1.3 meters above the riverbed in the Weimen reach.

Fig 4 and S1 Fig show that the cross-sectional mean vorticity strength was higher in the spawning reaches than in the non-spawning reaches and also illustrates the obvious discrepancy between the spawning and non-spawning reaches.

Based on a comprehensive analysis of the above data, the interval of cross-sectional mean vorticity preferred by *S*. *prenanti* in natural spawning grounds was 0.17 to 0.35 s^-1^, with higher values at the bottom of the central region of compound channels; the minimum value was 0.17 s^-1^, and the majority of values were greater than 0.26 s^-1^ in the spawning area. In addition, the area with the greatest vorticity values was approximately 1.3 m above the riverbed. This distribution of vorticity strength may provide a direction for future research.

### Test and Analysis Results

After performing the hypothesis test (see Eqs 10 and 11), the average rank of the vertical vorticity value was 4,079.8 for the spawning areas, including a total of 8,028 units, and the average rank of the vorticity value was 211.1 for the non-spawning areas, including a total of 6,973 units; we calculated Z = −439.7 and −439.7 < −1.645. Consequently, *H*_0_ was rejected. Therefore, we concluded that there were significant differences in the cross-sectional distributions between the spawning and non-spawning areas. This conclusion was consistent with the results of our field investigation, indicating that the vorticity strength selection of spawning *S*. *prenanti* may enhance our understanding of egg fertilization and breeding behavior.

### Vague-Set Similarity Model

The vague set model is an extension of the fuzzy set model, expanding the subordinate concept to the interval [0,1] so that the subordinate relationship of each element can be divided into two aspects of support and opposition. Its most prominent feature and advantage is that the vague set model can provide evidence in support or opposition and thus can be a more comprehensive expression of fuzzy information. The vague set model has been widely used in various branches of artificial intelligence, such as machine learning, decision analysis, knowledge acquisition, and pattern matching [59]. A collection of multi-indexes defined the hydraulic habitat for the spawning of *S*. *prenanti*. When each index is too large or too small, the habitat is not suitable for fish propagation. The hydraulic characteristics of the spawning grounds require additional fuzzy concepts for measurement and characterization. Therefore, a similar model was proposed based on vague sets of spawning habitat hydraulics of *S*. *prenanti*. In this study, the cross-sectional vorticity of the *S*. *prenanti* spawning hydraulic habitat was used as a new parameter to assess the similarity between rehabilitated spawning grounds (set*A*) and natural spawning grounds (set*B*). The default assumption was that the vorticity characteristics in the natural spawning reach are most suitable for fish spawning. If the restored spawning grounds are similar to the entire natural spawning grounds, then restoration will be effective.

The cross-sectional mean vorticity of the restoration target for *S*. *prenanti* was expressed as *U* = {*u*}. If the critical interval of the cross-sectional mean vorticity (*u*) in the restoration target is *A*, the degree of hesitation and the vague sets of the indicator *u* in *A* can be expressed as follows:

$$v_{A}(u)=\left\{\left(u,\left[t_{A}(u),1-f_{A}(u)\right]\right)\right\}$$ (12)

$$\pi_{A}(u)=1-t_{A}(u)-f_{A}(u)$$ (13)

where *t*_*A*_(*u*) is the subordinate degree to support *u* in the critical interval *A*, and *f*_*A*_(*u*) is the subordinate degree to oppose *u* in the critical interval *A*. *π*_*A*_ reflects neither support nor opposition to the extent of the hesitation. The cross-sectional mean vorticity of the natural spawning ground for *S*. *prenanti* was expressed as $\tilde{U}=\left\{\tilde{u}\right\}$. The degree of hesitation and the vague sets of the indicator $\tilde{u}$ in *B* were expressed as follows:

$$v_{B}(\tilde{u})=\left\{\left(\tilde{u},\left[t_{B}(\tilde{u}),1-f_{B}(\tilde{u})\right]\right)\right\}$$ (14)

$$\pi_{B}(\tilde{u})=1-t_{B}(\tilde{u})-f_{B}(\tilde{u})$$ (15)

Based on Li et al. [59] and Chen et al. [43], the vague-set similarity (*SIM*) between the restored spawning grounds and natural spawning grounds was expressed as follows:

$$SIM=T(u,\tilde{u})=1-\frac{|t_{A}(u)-t_{B}(\tilde{u})-\left(f_{A}(u)-f_{B}(\tilde{u})\right)|}{4}-\frac{|t_{A}(u)-t_{B}(\tilde{u})|+|f_{A}(u)-f_{B}(\tilde{u})|}{4}$$ (16)

The values of the cross-sectional mean vorticity in the Weimen spawning habitat were used as the appropriate interval of vague-set subordinate functions in combination with the vorticity value of non-spawning reaches to establish the computational boundary values. Table 3 presents the appropriate interval and boundary values of the cross-sectional mean vorticity in natural *S*. *prenanti* spawning grounds according to the analytical results of the vorticity distribution of high quality spawning habitat.

**Table 3:** Appropriate interval and boundary values of the cross-sectional mean vorticity in natural *Schizothorax prenanti* spawning grounds.

Name of index	Appropriate interval (*B*) (s^-1^)	Boundary value (s^-1^)
Cross-sectional mean vorticity	0.17–0.35	0.02–1.0

The vague values of the cross-sectional mean vorticity of the natural spawning grounds of *S*. *prenanti* were determined as follows:

$$\{\begin{array}{ll} v_{B}(x)=\left(t_{B}(x),1-f_{B}(x)\right)=(0,0) & x\leq 0.02\\ v_{B}(x)=\left(t_{B}(x),1-f_{B}(x)\right)=(\frac{x-0.02}{0.35-0.02},\frac{x-0.02}{0.2-0.02}) & \text{0.02}<x<0.2\\ v_{B}(x)=\left(t_{B}(x),1-f_{B}(x)\right)=(\frac{x-0.02}{0.35-0.02},\frac{1-x}{1-0.1}) & \text{0.2}\leq x<0.35\\ v_{B}(x)=\left(t_{B}(x),1-f_{B}(x)\right)=(\frac{1-x}{1-0.2},\frac{1-x}{1-0.35}) & \text{0.35}\leq x<1\\ v_{B}(x)=\left(t_{B}(x),1-f_{B}(x)\right)=(0,0) & x\geq 1 \end{array}$$ (17)

Based on previous restoration standards for fish spawning sites [35, 43], we constructed similarity evaluation criteria for the cross-sectional mean vorticity of the *S*. *prenanti* spawning grounds (Table 4).

**Table 4:** Similarity evaluation criteria for cross-sectional mean vorticity of *Schizothorax prenanti* spawning grounds.

SIM_vor_	[0.00–0.15)	[0.15–0.35)	[0.35–0.65)	[0.65–0.75)	[0.75–1.00]
Hydraulic habitat similarity	Extremely low	Lower	Low	General	Good

The effectiveness of rehabilitation measures was calculated as follows:

$$R=\frac{SIM_{after\_vor}\left(A,B\right)-SIM_{previous\_vor}\left(A,B\right)}{SIM_{previous\_vor}\left(A,B\right)}\times 100\%$$ (18)

where *R* is the rate of increase or decrease in *SIM*, *SIM*_*after_vor*_(*A*, *B*) is the vorticity similarity in the restored spawning area, and *SIM*_*previous_vor*_(*A*, *B*) is the vorticity similarity prior to restoration. A greater *SIM* value indicates greater similarity between the rehabilitated area and the natural spawning hydraulic habitat. After adoption of the related restoration measures, the rate of increase in *SIM* exceeded 5%, which should have a positive effect on the spawning grounds.

## Discussion

### Calculation Methods

Limited studies have examined the effects of vorticity characteristics on fish spawning grounds, though this subject is gradually attracting the attention of researchers. Complex flow patterns consisting of a variety of vortices are usually generated by irregular channel topography and provide unique habitats for numerous aquatic organisms. Because direct measurement of vorticities in natural rivers is currently impossible, numerical modeling of the flow structures surrounding complex topography is challenging and represents an important tool for aquatic habitat assessment [37].

The key to numerical simulation is a proper, precise and reliable method, which is directly related to the accuracy of the results. Using precise terrain measurements, this study applied the CLSVOF 3D model to simulate the hydraulic conditions of *S*. *prenanti* spawning grounds. The LS method is a popular interface tracking method for computing two-phase flows with topologically complex interfaces. This method is similar to the interface tracking method of the VOF model. However, with the LS method, a deficiency in preserving volume conservation was observed [53]. Because the VOF function (the volume fraction of a particular phase) is discontinuous across the interface, the weakness of the VOF method was in the calculation of the spatial derivatives. To overcome the deficiencies of the LS and VOF methods, a coupled LS and VOF approach was adopted to accurately calculate the curvature and the normal vector to the interface, whereas the VOF function was used to reconstruct the interface, guaranteeing conservation. Compared with other models, the CLSVOF model exhibits superior numerical stability, convergence and precision [52, 54, 55] and is particularly suitable for the simulation of mountain rivers with complicated terrain. In addition, compared with the 2D model, the 3D model can provide a much more accurate description of the heterogeneous velocity patterns favored by many aquatic species over a broad range of flows [37]. Characterization of 3D fluid motion is vital for fish that produce demersal eggs, such as *S*. *prenanti* and Chinese sturgeon. Spatial metrics can reveal coherent structures in the flow and provide a basis for characterizing the spatial coherence of 3D fluid motion, which is influenced by and influences the micro- and meso-scale morphology of streams [32]. Therefore, the introduction of the CLSVOF 3D model in this study effectively describes the ecological flow field and further broadens our understanding of the vorticity requirements for fish spawning, especially in situations in which the measured data flow fields are difficult to obtain. However, additional measurements are required to ensure the applicability of this method.

### Vorticity and Spawning

It is important to study vorticity for species where vorticity may influence their spawning process because a good understanding of the interactions between vorticity and spawning may help to protect endangered and endemic fish. Yang et al. [27–29] and Zhu et al. [33] studied this topic in fish spawning grounds by measuring the velocity field, and Wang et al. [30–32] studied this topic in fish spawning grounds through simulations with the Delft3D-Flow model. Their results suggested that vorticity metrics are sufficiently responsive and can describe the flow characteristics of spawning sites. Based on the results of the CLSVOF numerical simulations in this study, the Wilcoxon rank sum test was applied to examine discrepancies in the distributions of the hydraulic habitat factors of natural spawning areas to distinguish between spawning and non-spawning stretches. The application of the method in this study was effective and confirmed that significant differences exist in the vertical vorticity distributions between spawning and non-spawning reaches. Thus, vorticity may play a significant role in the spawning success of some types of fish, possibly as a result of natural selection in biological evolution [30], which is consistent with the aforementioned studies.

Three factors are widely acknowledged and discussed in relation to the effect of vortex motion on the spawning behavior of fish. The first and perhaps most important factor is that vortex motion can increase the mixing intensity of sperm and eggs, thereby increasing the fertilization rate. This is a common explanation by biological scientists [15–17], although it is not well understood. Numerous studies have focused on the relationship between vorticity and fertilization rates, such as Crimaldi and Browning [60], who used numerical simulations of simple vortex flows to propose a mechanism for the turbulent enhancement of broadcast spawning efficiency in which instantaneous turbulent processes might promote short-term coalescence between high-concentration filaments of eggs and sperm, significantly enhancing the average fertilization rate. Crimaldi et al. [61–63] then investigated a series of reactive advection-diffusion problems motivated by the ecological mixing process and demonstrated the ability of structured flow fields to impart spatial correlations into a pair of initially distant scalars (surrogates for eggs and sperm), thereby enhancing reactions or fertilization rates. As concentrations of eggs and sperm begin to coalesce, fertilization rates increase rapidly until reaching a peak. In addition, simulated vortex stirring shortens the time required for peak fertilization to occur [63]. Yang et al. [27–29], Wang et al. [30–32] and Zhu et al. [33] calculated the absolute values of vorticity along river cross-sections or planes using field data and reported a strong link between vorticity strength and the fertilization rates of fish. Thus, indirect but critical evidence has been obtained that larger vorticities increase fertilization rates. However, an extremely high vorticity can lead to egg wash-off or predation by other fish, and egg incubation cannot be effectively protected [30, 32]. Hence, determining the upper limit of vorticity in spawning grounds is just as important as determining the lower limit. Second, vortex motion can strengthen the mixing and convective exchange of matter and energy and supply sufficient oxygen and constant temperatures to fertilized eggs, thus resulting in an enhanced hatching rate [15, 16, 18, 40]. Third, the vortex motion facilitates the spread and adhesion of fertilized eggs in gravel and rocks [15–18, 25, 26], which leads to a higher survival rate.

*S*. *prenanti* is a mountain-river dwelling fish with a preference for high dissolved oxygen content and sand or gravel bottoms, and it lays viscous demersal eggs with external fertilization. Spawning usually occurs from March to May at temperatures of 9–19°C, usually in rapids and shoals with rolling and upwelling water [64, 65]. A vortex is a prominent characteristic of ‘rolling and upwelling water’ and the source of the mixing effect [48]. For fish that practice external fertilization, a high degree of turbulent flow is often preferable for reproduction, and mating behavior will often not occur until the flow reaches a certain degree of disorder [27]. In this research, the average cross-sectional vorticity of spawning areas was 2.64 times that of non-spawning areas. Thus, vortices with higher vorticity values may provide increased contact between eggs and sperm, resulting in enhanced fertilization rates. In addition, flow aeration and heat mixing driven by the vortex motion provide suitable physical conditions for the spawning of *S*. *prenanti* and govern fertilization success.

Terrain factors also affect the regional distribution of streamwise vorticity in *S*. *prenanti* spawning grounds. However, greater vorticity was generally observed at the bottom of shallow water compound channels, and the optimum spawning depth was 1.3 m above the riverbed. The vorticity strength was significantly greater in relevant sections of the *S*. *prenanti* spawning areas than in the transition and non-spawning areas, and vorticity was primarily distributed at the riverbed, which coincided with long-term survival of fish on the river bottom. The vorticity levels varied with variations in channel topography. The shape of the cross-sections gradually changed in transition areas from compound shapes to V-shapes, although all the cross-sections were V-shaped in non-spawning areas. From the spawning to non-spawning areas, the magnitude of vertical vorticity of the river section decreased as the velocity decreased, the river widened, and the water depth increased. The transverse distribution of vorticity in *S*. *prenanti* spawning areas was uneven, with large values in the middle and small values at both edges. Concentrated high velocity was observed primarily at the bottom. Overall, the range of cross-sectional mean vorticity strength was approximately 0.17 to 0.35 s^-1^ for *S*. *prenanti* spawning sites, although *S*. *prenanti* spawning sites might be distributed over the areas with vorticity strengths greater than 0.26 s^−1^. Yang et al. [27, 28] concluded that the range of cross-sectional mean vorticity strength was approximately 0.27 s^-1^ to 0.85 s^-1^ for Chinese sturgeon; however, Chinese sturgeon spawning sites were primarily distributed over the area with vorticity strengths greater than 0.4 s^-1^. The cross-sectional vorticity characteristics varied with fish category and living area; therefore, we speculate that larger fish eggs require greater vorticity strength because the diameters of Chinese sturgeon and *S*. *prenanti* eggs are variable (2.0–4.8 mm for the former [27–29] and 2.5–3.4 mm for the latter [65]), although their eggs are similarly sinking and viscous. In addition, Chen et al. [43] assumed that *S*. *prenanti* prefers to spawn in areas with horizontal vorticity of 0.1–0.2 s^-1^, whereas Wang et al. [30] suggested that Chinese sturgeon prefer spawning areas where the horizontal mean vorticity is less than 1.0×10^−3^ s^-1^. The vertical and horizontal vorticities characterize the rotational motion intensity along the cross section and horizontal plane, respectively, and both are used to describe the frequencies of small eddies within a certain range. Greater vorticity values indicate a greater number of eddies and more turbulent flow fields, although scant information is available regarding the variable effects among different vorticity components. However, fish appear to favor vertical vorticity more than horizontal vorticity; thus, it is hypothesized that the vertical component of vorticity may account for a greater proportion of the ecological effects of vorticity for fish that lay demersal and viscous eggs. Hence, we recommend further research to establish comprehensive spawning preferences for various fish and identify the roles of different vorticity components.

Cross-sectional vorticity may plays a crucial role in the spawning grounds of fish, particularly for fish (such as *S*. *prenanti*) that lay sinking and viscous eggs, because cross-sectional vorticity can more clearly describe vertical variations in the flow field complexity and increase the blending intensity of sperm and eggs to increase the rate of fertilization. Therefore, in addition to its engineering and scientific applications, cross-sectional vorticity is an important factor that should be considered in the rehabilitation of hydraulic habitats that serve as spawning grounds for fish.

### Application of Vorticity Characteristics

If a specific spatial metric is determined to be biologically significant, then it can be incorporated into habitat suitability criteria for that particular organism [21]. The vorticity similarity vague-set model was proposed to provide comparisons among spawning habitat features between restored reaches and natural reaches that are hydraulically similar or different and explain how these differences might reflect aquatic health and the effectiveness of measures to rehabilitate reaches.

The ultimate purpose of this paper was to offer targets and standards for the ecological restoration of spawning grounds for *S*. *prenanti* or other similar fish. To determine whether the reach of river had the hydraulic characteristics required of spawning grounds, a number of concepts were measured and characterized. Similarity measurements with vague sets are widely used in fuzzy inference, decision analysis, cluster analysis, and pattern recognition, among others [66, 67], and vague sets were used first to construct a similarity model of the cross-sectional vorticity in this study. The parameters of the model were the variables in the index system of the spawning grounds of *S*. *prenanti*, and the hydraulic characteristics of the natural spawning grounds provided the appropriate ranges. The results of this study should be beneficial to verify the designs and effects of ecological restoration.

The spatial hydraulic metrics proposed here are among the many potential metrics that could be employed for evaluations of spawning conditions. However, Chen et al. [43, 44] established a hydraulic system composed of three levels and six indexes to provide correlations among the spawning grounds of *S*. *prenanti* and obtained a range of each index, which indicated an average depth of 1.2–1.8 m, an average velocity of 1.3–2.0 m s^-1^, a velocity gradient of 0.09~0.21 s^-1^, a kinetic energy gradient of 0.09–0.32 J kg^-1^ m^-1^, a Froude number of 0.3–0.5, and a horizontal mean vorticity of 0.1–0.2 s^-1^. A similarity model of hydraulic habitats giving equal weight to the six indexes was proposed, and the results of this study can therefore be directly incorporated into the original system as important supplementary data to help form a more complete framework to increase the efficacy of restoration and protection measures for the spawning grounds of *S*. *prenanti* and other similarly endangered species in the region.

Finally, this study has limitations, such as the lack of direct validation data, lack of accurate density data for fish eggs and sperm, difficulty of finding more typical spawning grounds, failure to distinguish among biological differences for the hydraulic variables, and insufficient ability to identify internal connections and independence of various hydraulic metrics. Nonetheless, global hydropower engineering is concentrated in China, specifically in the southwestern regions. The ecological problems caused by engineering construction, such as damage to riverine habitats and reduced biological diversity, have not garnered sufficient attention in the past, yet government agencies and scholars are working to compensate for such adverse outcomes. Although the successful restoration of the spawning grounds of *S*. *prenanti* has not been reported, we provide a tentative theoretical basis for the recovery of damaged *S*. *prenanti* spawning grounds. Certainly, further comprehensive and holistic characterizations of the spawning requirements of *S*. *prenanti* must be conducted. Such investigations will be the focus of our future work, to determine the best and most appropriate uses of various metrics and to evaluate the coefficients and weight matrices of these metrics.

## Conclusions

In this research, the CLSVOF model was applied to simulate the hydraulic habitat of natural *S*. *prenanti* spawning grounds in the Minjiang River. The cross-sectional vorticity was used to assess the hydraulic environment of spawning grounds. Statistical analyses revealed that vorticity strength was significantly greater in relevant sections of *S*. *prenanti* spawning areas than in transition and non-spawning areas, and vorticity was primarily distributed at the bottom of the riverbed. The average cross-sectional vorticity of spawning areas was 2.64 times that of non-spawning areas. *S*. *prenanti* had a tendency to spawn in areas with greater vorticity values, and the range of cross-sectional mean vorticity was 0.17 s^-1^–0.35 s^-1^ in observed perennial areas with concentrated eggs and sperm. These findings may confirm the cross-sectional vorticity strength selection of spawning *S*. *prenanti*, facilitate the development of strong spatial correlations between eggs and sperm, and indicate suitable physical conditions for fertilized eggs, which will improve our understanding of egg fertilization and breeding behavior.

Finally, the extracted cross-sectional mean vorticities of *S*. *prenanti* habitats were applied to a vague-set similarity model, and the results improved our current hydraulic habitat index system; with additional observations and validation, they have the potential to influence practical processes in ecological restoration.

This study provides further insight into the hydraulic requirements of *S*. *prenanti* for spawning. To determine the best and most appropriate use of various metrics as well as the evaluation coefficients and weight matrices of these metrics, future work must focus on typical spawning and provide meticulous observation of spawning behavior.

## Supporting Information

S1 Fig Changes of cross-sectional mean vorticity of the whole section in the Weimen reach. (TIF)
