# Supplementary figures and images for: Characteristics of the Cross-Sectional Vorticity of the Natural Spawning Grounds of Schizothorax prenanti and a Vague-Set Similarity Model for Ecological Restoration
Source: PLoS One. 2015 Aug 28;10(8):e0136724. doi: 10.1371/journal.pone.0136724 (PMC4552946; doi:10.1371/journal.pone.0136724)

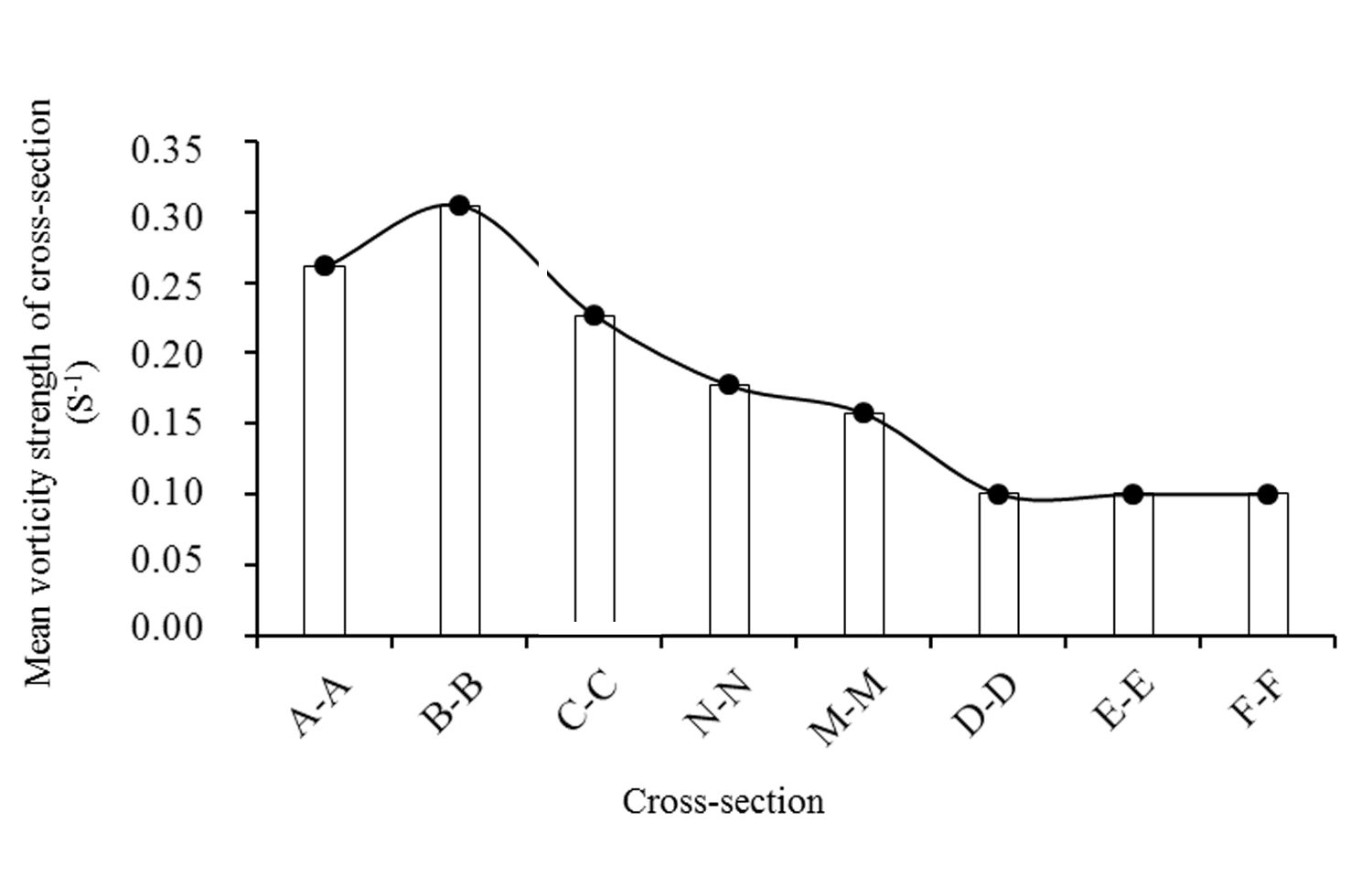

Supplement: S1 Fig — (TIF) [file pone.0136724.s001.tif]
